# Comparing low-field cryogenic nuclear relaxation of hyperpolarized diamond and silicon particles

**DOI:** 10.1038/s41598-025-33130-3

**Published:** 2025-12-27

**Authors:** Gevin von Witte, Mohammed M. Albannay, Matthias Ernst, Sebastian Kozerke

**Affiliations:** 1 Institute for Biomedical Engineering, University and ETH Zurich, 8092 Zurich, Switzerland; 2 Institute of Molecular Physical Science, ETH Zurich, 8093 Zurich, Switzerland

**Keywords:** Diamond, Dynamic nuclear polarization (DNP), Relaxation, Field-cycling, Silicon, Applied physics, Quantum physics, Biomedical engineering, Chemical physics

## Abstract

We report on field cycling experiments with hyperpolarized diamond and silicon particles between 10 mT and 3.4 T at temperatures below 10 K. Diamonds with approximately 54 ppm defects, of which around 58% were P1 centers, were hyperpolarized by continuous-wave dynamic nuclear polarization (DNP) at 3.4 T. For fields above 200 mT, the ^13^C relaxation in diamond was measured to be nearly independent of the magnetic field. At around 200 mT, the field dependence changed and $T_1$ was approximately proportional to the field strength. For example, the relaxation time decreased approximately threefold by reducing the main magnetic field from 200 mT to 75 mT. The ^13^C relaxation was measured to be independent of the DNP polarization time and nuclear hyperpolarization levels. In contrast, the relaxation of hyperpolarized silicon was found to be independent of the field strength down to a few mT, despite a relatively short time for DNP build-up. The results suggest that magnetic fields greater than approximately 200 mT are required for hyperpolarized diamonds with several ppm of (nitrogen) defects to ensure sufficiently long relaxation times.

## Introduction

Spin hyperpolarization refers to a non-equilibrium state of spin polarization exceeding the thermal equilibrium Boltzmann polarization. The hyperpolarized state relaxes back to thermal polarization on a time scale characterized by the spin-lattice relaxation time $T_1$, after termination of the hyperpolarization process. For applications using the hyperpolarized state, sufficiently long $T_1$ relaxation times are required. Moreover, it has been shown that $T_1$ relaxation also limits the achievable polarization levels as it counteracts the hyperpolarization process^1,2^.

Hyperpolarized ^13^C in diamond has been used to study nuclear relaxation^3–5^ and quantum many-body systems^6,7^, extend electronic coherence times^5,8^ with possible applications in hyperpolarized nuclear magnetometry^9^ and nuclear spin qubits^10,11^, generate entanglement between nitrogen vacancy (NV) centers^12^, and, act as an imaging agent for magnetic resonance imaging (MRI)^13–18^. ^13^C nuclear spins in diamond can be hyperpolarized by brute force (cooling to conditions with high thermal nuclear polarization)^13^, continuous wave (CW) dynamic nuclear polarization (DNP)^3,13–17,19–22^, pulsed DNP-like nuclear orientation via electron spin-locking (NOVEL)^23^ or DNP following optical hyperpolarization of NV centers^4–7,18,24–29^. Nuclear relaxation in diamond has been studied at low^30^ and high^4,31–33^ magnetic fields, for ^13^C enriched diamonds^34^ and hyperpolarized diamonds^3,4,16^. However, none of these studies has combined low temperatures with magnetic fields below 100 mT. Such low magnetic fields are encountered during optical hyperpolarization with NV centers^4–7,18,25–27,29^ or during transfer of hyperpolarized samples from the polarizer to an MRI scanner^15,16^.

In this work, we study relaxation of diamond microparticles in decreasing magnetic fields ranging from 3.4 T to 10 mT^15,22^ at temperatures equal or below 10 K after hyperpolarization with CW DNP involving P1 centers of the microparticles^22^ at 3.4 T. The chosen range of magnetic fields and temperatures is, e.g., encountered during the transfer of an hyperpolarized sample to an MRI scanner, as the hyperpolarized sample needs to be lifted out of the liquid helium bath of the hyperpolarizer magnet. Since both hyperpolarized diamond and silicon particles have been proposed for MRI applications, we compare the field dependence of relaxation in diamond and silicon particles^35–39^.

## Methods

**Figure 1:**
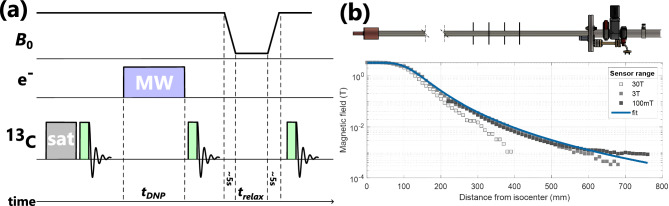
(**a**) Experimental procedures involving radio-frequency (RF) pulsing on the ^13^C nuclei, microwave (MW) irradiation of the electrons for DNP and magnetic field ($B_0$) cycling. After saturation of the ^13^C nuclear spins by RF pulses and verification of its effectiveness, DNP is applied by MW irradiation of the electrons for duration $t_\textrm{DNP}$. The DNP signal is measured on the ^13^C nuclei. Shuttling of the sample to the target magnetic field took around 5 s. After $t_\textrm{relax}$ at the target magnetic field, the sample is shuttled back to the iso-center within around 5 s for detection of the remaining ^13^C signal. (**b**) Design of the probe and the magnetic field profile of the magnet. The overmoded cavity containing the sample during DNP is to the left (at the magnetic iso-center with 3.4 T). Field cycling experiments over more than three orders of magnetic field strength are possible. The magnetic field was measured as a function of distance from the iso-center using different magnetic field sensors to account for their different sensor ranges.

### Samples

High-pressure high-temperature (HPHT) synthesized mono-crystalline diamonds with an average particle size of $10 \pm 2$ µm were purchased from Microdiamant AG (Switzerland). The defect concentration of the diamonds was estimated to around 54 ppm with 58% of the defects being P1 centers^22^. Diamonds were used without further treatment.

For comparison, $>99$% ^29^Si enriched particles (Isoflex, Russia) were utilized as reference. The particles were previously used for thermal equilibrium experiments^35–39^, offering relatively large NMR signals owing to the high ^29^Si spin density. The defect concentration and particle size of the silicon particles have not been characterized. Section S2 and especially Fig. S1 of the Supplementary Information, report and discuss the DNP build-up and DNP profile of the ^29^Si enriched particles in comparison to recently published natural abundance silicon nanoparticles hyperpolarized under similar experimental conditions^37,39^.

### Dynamic nuclear polarization

The DNP measurements were performed using a home-built polarizer operating at 3.4 T based on a Bruker 300 wide-bore shielded magnet and equipped with an OpenCore NMR^40–42^ spectrometer. A VDI (Virginia Diodes Inc., USA) microwave source was used to output 400 mW. The cryostat insert was based on Ref.^43^ and is depicted in Fig. 1b. The sample vial was tethered by a thin thermoplastic tube, inserted through a gate valve into the sample tube with an overmoded cavity at the bottom. The overmoded cavity contained the MW waveguide outlet for DNP and an Alderman-Grant coil for NMR detection.

### Field cycling experiments

NMR signals were recorded using radio-frequency (RF) pulses with a flip angle of around 8-10° in all the field-cycling experiments. The procedure of the field cycling experiments is depicted in Fig. 1a. The nuclear spins (^13^C or ^29^Si) were first saturated with a train of large flip angle RF pulses. The saturation of all detectable nuclear polarization was verified with a read-out RF pulse. Subsequently, microwave (MW) irradiation was applied to create nuclear hyperpolarization with DNP for a duration $t_\textrm{DNP}$. Thereafter, the MW was switched off, the nuclear signal measured, the sample shuttled to the target field (cf. field profile along the *z*-direction of the magnet in Fig. 1b), kept at the target field for a duration $t_\textrm{relax}$ and finally shuttled back to the magnetic iso-center for read-out. The shuttling was performed manually using the thermoplastic tube attached to the sample vial inside the sample tube. The up and down shuttling each took $t_\textrm{shuttle} \approx 5$ s (cf. Fig. 1a).

After turning off the MW irradiation, a waiting time $t_\textrm{wait} \approx 2$ s was required for signal acquisition before shuttling could begin. In a similar manner, $t_\textrm{wait} \approx 2$ s between completion of the shuttling and final ^13^C signal detection was required for verification of the sample position and signal acquisition. Thus, the whole shuttling process took $t_\textrm{cycle} = 2t_\textrm{wait} + 2t_\textrm{shuttle} + t_\textrm{relax}$. The waiting time involved relaxation at the nominal magnetic field and is included in the analysis.

**Figure 2:**
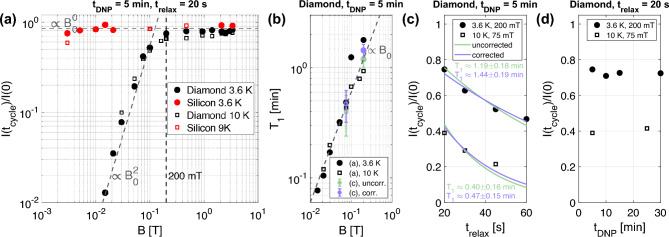
(**a**) Fraction of the relative signal intensity after the field cycling with respect to the initial DNP signal. Diamond and silicon particles were hyperpolarized for $t_\textrm{DNP} = 5$ min. The sample was allowed to relax for $t_\textrm{relax} = 20$ s at the magnetic fields ($t_\textrm{cycle} \approx 34$ s). (**b**) (black) Extracted $T_1$ times from the low field part of diamond at 3.6 K and 10 K as shown in (a). The $T_1$ times were extracted from panel (**a**) with an iterative algorithm described in the main text and Sec. S1 of the Supplementary Information. To assess the validity of the $T_1$ times determined using the iterative algorithm, the $T_1$ times obtained from mono-exponential fits to the data in panel (**c**) are displayed (green and purple). (**c**) Relative signal intensity as a function of the relaxation delay. For the “uncorrected” fits, the relative signal for $t_\textrm{relax} = 0$ s was set to one, implicitly assuming the absence of a signal intensity loss by the shuttling. For the “corrected” fits, the signal intensity loss from shuttling as estimated by the iterative algorithm in panel (**b**) is subtracted from the assumed $t_\textrm{relax} = 0$ s signal intensity, e.g. 0.91 for 3.6 K, 200 mT and 0.86 for 10 K, 75 mT. (**d**) Dependence of relative signal intensity on DNP time for diamond. For all experiments, except for those whose data are presented in panel (**c**), only a single measurement could be performed per condition and hence measurement uncertainty could not be assessed.

To convert the measured signal intensity loss into a spin-lattice ($T_1$) relaxation time for a given static magnetic field strength (height), the relaxation of the fields (heights) traversed during the shuttling to the target magnetic field (height) needs to be considered. This was calculated iteratively starting with the highest magnetic field (in the magnetic iso-center) for which a mono-exponential $T_1$ relaxation was derived from the signal intensity loss. Between heights for which the relaxation was measured (data points of Fig. 2a for higher magnetic fields/ lower heights), the relaxation rate was linearly interpolated (assuming constant velocity of sample container). Numerical summation over all the relaxation contributions at the different heights traversed yielded the signal intensity loss, which was subtracted from the total signal intensity loss ($I(t_\text {cycle})/I(0)$) before the mono-exponential $T_1$ time was calculated. This is summarized in the commented pseudo-code given in Sec. S1 of the Supplementary Information.

## Results

The normalized integrated intensity of the NMR signals after field cycling ($t_\textrm{DNP} = 5$ min, $t_\textrm{relax} = 20$ s) for diamond and silicon are shown in Fig. 2a. For silicon at 3.6 K and 9 K, the signal intensity appears to be independent of the magnetic field down to 3-5 mT. Above around 200 mT, the intensity of the diamond signal is found to be independent of the magnetic field. Below 100-200 mT, the remaining signal intensity has an approximately quadratic dependence on the magnetic field, corresponding to a linear dependence on the $T_1$ time as shown in Fig. 2b and described below. After 20 s at 15 mT and 3.6 K, close to no signal intensity was detected. Increasing the temperature to 10 K gives the same results with lower initial signal, which results in the absence of a detectable signal at around 20 mT.

The $T_1$ values in Fig. 2b are computed with an iterative algorithm described in the Methods and Sec. S1 of the Supplementary Information based on the results shown in Fig. 2a. To stabilize the iterative algorithm, the relaxation above 200 mT was assumed to be field-independent. Specifically, the relaxation times at all the heights up to 200 mT were computed with the averaged signal intensity loss of all data points $\ge 200$ mT. The signal intensity loss by the $T_1$ relaxation at the intermediate magnetic fields during the shuttling is included in the analysis with a linear interpolation time slicing algorithm (cf. Methods and Sec. S1 of the Supplementary Information). This reduced signal intensity loss is used to calculate a mono-exponential $T_1$ as shown in Fig. 2b, which shows $T_1 \propto B_0$ below approximately 200 mT.

To validate the relaxation times obtained from the measurement shown in Fig. 2a with $t_\textrm{relax} = 20$ s, Fig. 2c shows the signal intensity for different relaxation delays $t_\textrm{relax}$ with the signal intensity monotonically decreasing for longer relaxation delays. The fitted $T_1$ times of the decreasing signal with $t_\textrm{relax}$ are in good agreement with the $T_1$ times extracted from Fig. 2a as shown in Fig. 2b. To fit the $T_1$ times from relaxation delays, it needs to be considered that the shuttling to the target field causes a signal intensity loss. This can be included in the fitting as a reduced amplitude of the mono-exponential fit with the starting values of these “corrected” fits based on the iterative algorithm used for Fig. 2b as described above and in Sec. S1 of the Supplementary Information. Similar results of the “uncorrected” and “corrected” fits facilitate the results obtained with the iterative algorithm shown in Fig. 2b.

Fig. 2d shows that the relative signal intensity is independent of the initial DNP duration $t_\textrm{DNP}$. Longer $t_\textrm{DNP}$ corresponds to a higher initial nuclear polarization (signal intensity) at the beginning of the field cycling experiment (cf. Ref.^22^).

## Discussion

This work shows that $T_1$ relaxation of hyperpolarized ^13^C in diamond microparticles scales approximately proportional to the field strength below 200 mT, potentially limiting applications in which the hyperpolarized particles need to be lifted out of the hyperpolarizing magnet. The $T_1$ relaxation times as shown by the black points in Fig. 2b were computed with a mono-exponential ansatz based on the signal intensity loss. This approach involves two major simplifications. First, only two measurements (signal before and after shuttling) were used to compute the mono-expontial $T_1$ instead of multiple data points. The mono-exponential fits of the relaxation delay as shown in Fig. 2c served to validate the approach with the different $T_1$ estimates in Fig. 2b. Second, the polarization dynamics in diamond is not mono-exponential but rather characterized by a stretched exponential as discussed in Ref.^22^. In Sec. S3 of the Supplementary Information, a direct comparison of mono-, bi- and stretched exponential fits is provided. While the bi- and stretched exponential fit are sufficiently accurate, the mono-exponential fit performs only reasonably well to quantify the polarization dynamics.

Based on the measured stretched exponential decay ($T_1$) time of diamond at 3.4 T and 3.5 K of $7.4 \pm 0.4$ min with a stretch exponent of $0.71 \pm 0.08$^22^, a signal intensity loss of around 16% during the first 34 s after switching off the MW ($t_\textrm{cycle} = 2t_\textrm{wait} + 2t_\textrm{shuttle} + t_\textrm{relax} = 2 \cdot 2 + 2 \cdot 5 + 20~\textrm{s} = 34$ s) is expected, which is in agreement with the high field ($>200$ mT) results shown in Fig. 2a.

Room temperature relaxation times of natural abundance ^13^C diamond can range from minutes to hours^3,22,30–33^ and have been explained through a mixture of electron flips or triple spin flips^3,4,30,32,33,44^ with exponents of the magnetic field dependence varying between roughly 0.5 and 2. For fields below 200 mT and temperatures below 10 K, we find an approximately linear dependence of the $T_1$ relaxation time on the magnetic field (cf. Fig. 2b). Such a linear dependence of the $T_1$ time on the magnetic field below around 100-200 mT^45,46^ has been reported for proton and carbon DNP of 15 mM trityl with pyruvate at cryogenic temperatures. Assuming that the trityl density (unpaired electrons) $n_\textrm{e} = 15$ mM is homogeneously distributed throughout the sample, this corresponds to an average distance between electrons of $r_\mathrm {e-e} = n_\textrm{e}^{-1/3} \approx 4.8$ nm after conversion from mM to number of unpaired electrons per unit volume. This average distance is similar to our diamond sample with distances between defects of around 4.7 nm assuming a homogeneous distribution^22^.

The linear field dependence of the (hyperpolarized) nuclear relaxation at low magnetic fields, e.g. below 200 mT, in diamond and organic compounds might point to a common and eventually more widely occurring origin. The nuclear polarization ($P_\textrm{n}$) dynamics in spin systems containing (dipolar) coupled electrons on a macroscopic level can be described by Eq. 8.54 of the book from Wenckebach^47^

1 $$\begin{aligned} \frac{\partial P_\textrm{n}}{\partial t} = -\frac{1}{2} \pi \frac{N_\textrm{e}}{N_\textrm{n}} \frac{A^2D^2}{\omega _n^2} \int _{-\infty }^\infty \text {d}\omega ~ g(\omega ) g(\omega -\omega _\textrm{n}) ~ \left[ 1 - P_{\textrm{e}}(\omega ) P_{\textrm{e}}(\omega -\omega _\textrm{n}) \right] ~\left[ P_\textrm{n} -\frac{P_{\textrm{e}}(\omega ) - P_{\textrm{e}}(\omega - \omega _\textrm{n})}{1 - P_{\textrm{e}}(\omega ) P_{\textrm{e}}(\omega - \omega _\textrm{n})} \right] \end{aligned}$$

with $N_\textrm{e}$ ($N_\textrm{n}$) the number of electron (nuclear) spins, $D^2$ ($A^2$) describe the electron dipolar (hyperfine) couplings of the spin system (cf. Ch. 8 of the book by Wenckebach^47^ for more details) and $g(\omega )$ is the electron spectral line shape. $P_e(\omega )$ is the electron polarization at a given frequency and $\omega _\textrm{n}$ the nuclear Larmor frequency given by the external magnetic field $B_0$. The absence of MW irradiation during the shuttling leads to an equal electron polarization across the electron spectral line. Moreover, for low magnetic fields, the thermal electron polarization is small and for simplicity can be assumed to be zero. Thus, we can ignore the electron polarization terms, which are part of the integral. This leaves us with

2 $$\begin{aligned} \frac{\partial P_\textrm{n}}{\partial t} \approx -\frac{1}{2} \pi \frac{N_\textrm{e}}{N_\textrm{n}} \frac{A^2D^2}{\omega _n^2} \int _{-\infty }^\infty \text {d}\omega ~ g(\omega ) g(\omega -\omega _\textrm{n}) ~ P_\textrm{n} \end{aligned}$$

The differential equation gives a mono-exponential solution. The $T_1$ time is given by the integral and prefactors of Eq. (2). The linear dependence of $T_1$ on $B_0$ arises from the $\omega _\textrm{n}^{-2} \propto B_0^{-2}$ scaling and the notion that the electron spectral line shape in many cases is linearly dependent on the field strength, e.g. *g*-factor anisotropy. A linear dependence of the electron line width on the magnetic field was experimentally observed for 15 mM trityl^48^. The question of *g*-factor anisotropy in diamond is discussed in Sec. S4 of the Supplementary Information. A $T_1 \propto B_0$ scaling at low magnetic fields without MW irradiation in spin systems with sufficient electron dipolar couplings at cryogenic temperatures might be observed in a wider range of materials. The onset of $T_1 \propto B_0$ is attributed to the unpaired electron density, supported by room-temperature relaxation experiments with diamond observing a similar change of field dependence as in this work^4^.

An alternative explanation for the observed field dependence of the $T_1$ involves the experimental set-up with the top of the cryostat flange close to room temperature and the inner bottom of the cryostat at or close to liquid-helium temperatures. Lifting the sample to lower magnetic fields corresponds to higher temperatures. The sample contains several tens of milligrams of diamond particles inside a polymer sample container (1 mm wall thickness), which itself sits inside a polymer sample holder with at most the bottom of the sample container in direct contact with the cryostat atmosphere. This design should provide sufficient insulation of the sample during the shuttling and relaxation at lower magnetic fields where it is immersed in helium gas at temperatures above the temperature at the magnetic center (cf. Fig. 2). This is supported by the absence of substantial pressure increases upon lowering the sample back into the coil. Inserting the sample from room temperature within 10-20 s into the liquid helium results in a pressure increase inside the cryostat by 0.1-0.2 bar or more.

The observed accelerated ^13^C relaxation in our sample at lower fields ($\le 0.1$ T) might rationalize polarization levels in diamond hyperpolarized through NV centers. For high defect concentrations, the hyperpolarization build-up only takes a few hundred seconds^24–26,28^, while for low defect concentrations this takes tens of minutes^29^. Speaking in terms of a rate-equation model of hyperpolarization^1,2^, a high defect concentration causes a large ^13^C relaxation rate constant (short $T_1$ time) at low fields as observed in this study and Ref.^4^. In contrast, for low defect concentrations, the relaxation rate constant is small but the hyperpolarization injection rate decreases by a similar amount compared to the relaxation rate, resulting in similar steady-state polarization levels around 5%^24,25,29^ only with longer $T_1$ relaxation and build-up times for low defect concentrations.

For silicon, the situation is different. The measured signal is independent of the magnetic field down to a few mT (Fig. 2a). The low relaxation of silicon in our experiments was expected based on Ref.^49^, which investigated the nuclear relaxation of hyperpolarized silicon particles in detail. The different cryogenic low field relaxation of diamond and silicon are likely related to the defect distribution in the particles. The paramagnetic defects in diamond are mostly bulk defects which lead to nearly all nuclear spins experiencing hyperfine couplings of a few kHz^22^. In silicon, the defects are close to the surface of the particles, creating a core-shell situation with the polarization in the core spatially separated from the defects^37,39,49,50^.

## Conclusions

Exposure of hyperpolarized diamond particles to magnetic fields below approximately 200 mT should be avoided, if high polarization levels are required for further applications, e.g., hyperpolarized magnetic resonance imaging. Hyperpolarized silicon particles appear rather insensitive to varying magnetic fields^49^, making them better candidates for hyperpolarized MRI.

## Supplementary Information

Supplementary Information.

## Data Availability

Experimental data together with a Matlab script can be found under https://doi.org/10.3929/ethz-b-000719925.
