# Supplementary material for: Comparing low-field cryogenic nuclear relaxation of hyperpolarized diamond and silicon particles
Source: Sci Rep. 2025 Dec 27;16:3202. doi: 10.1038/s41598-025-33130-3 (PMC12830836; doi:10.1038/s41598-025-33130-3)
Supplement: Supplementary file 1 — Supplementary Information. [file 41598_2025_33130_MOESM1_ESM.pdf]

Supplementary Information: Comparing low-field cryogenic nuclear relaxation of hyperpolarized diamond and silicon particles

Gevin von Witte[1,2], Mohammed M. Albannay[1,2], Matthias Ernst[2], and Sebastian Kozerke[1,*]

[1]Institute for Biomedical Engineering, University and ETH Zurich, 8092 Zurich, Switzerland
[2]Institute of Molecular Physical Science, ETH Zurich, 8093 Zurich, Switzerland
[*]kozerke@biomed.ee.ethz.ch

Contents

S1 Pseudo-code to convert measured signal intensity loss into T_1 2
S2 DNP experiments of the silicon sample 2
S3 Exponential fits of diamond 3
S4 DNP in diamond 4
References 4

S1 Pseudo-code to convert measured signal intensity loss into T_1

```
R_1 ← zeros(n)                                   ▷ R_1 is the relaxation rate; n is the number of data points (magnetic fields/ heights) measured
S_rel ← [I(t_cycle)/I(0)]                        ▷ Simplify notation
R_1(1) ← S_rel(1)/(2t_wait + 2t_shuttle + t_relax)
IntensityLossShuttling ← ones(n) · R_1(2t_wait)  ▷ Signal intensity loss by waiting at iso-center before and after shuttling
IntensityLossShuttling(1) ← R_1(1) · (2t_wait + 2t_shuttle)
τ ← 0.001 s                                      ▷ τ is the time step of the time slicing
for 2 ≤ m ≤ n do                                 ▷ Index shift due to relaxation at the iso-center as first element
    for 2 ≤ i ≤ m − 1 do                         ▷ Calculate signal intensity loss from shuttling up to last measured magnetic field/ height
        k ← round( (height(i)−height(i−1))/height(m−1) · t_shuttle/τ )   ▷ Set number of interpolation steps
        for 1 ≤ j ≤ k do
            IntensityLossShuttling(m) ← IntensityLossShuttling(m) + ( R_1(i−1) + (R_1(i)−R_1(i−1))/k · j ) τ
        end for
    end for
                                                 ▷ Subtraction of signal intensity losses from shuttling and computation of relaxation rate at target magnetic field/ height
    k ← round( (1 − height(m−1)/height(m)) · t_shuttle/τ )
    R_1(m) ← ( −log(S_rel(m−1)) − 2IntensityLossShuttling(m) − 2R_1(m−1)τ(k−1)/2 ) / ( t_relax + τ(k+1)/2 )
                                                 ▷ τ(k ± 1)/2 arise from linear interpolation between the relaxation at the previous and currently measured fields, which means the sample spends virtually half the time of the last shuttling step at each of the fields with their respective relaxation rates
end for
T_1 ← 1/R_1
```

S2 DNP experiments of the silicon sample

Fig. S1a, b shows the DNP build-up of the $> 99\%$ ^{29}Si particles around 3.5 K at 3.4 T and 7 T. In both cases, the build-up slightly deviates from a mono-exponential fit, while a bi-exponential fit describes the measured data very well. At 7 T, the thermal equilibrium ^{29}Si signal was measured and polarization levels of around 1.4% have been achieved in the silicon sample with DNP. Fig. S1c shows the DNP profile of the $> 99\%$ ^{29}Si particles around 3.5 K at 7 T

The measured DNP enhancements (polarization) and MW frequency with the highest DNP enhancement are comparable to natural abundance silicon nanoparticles measured in the same experimental set-up[1,2], indicating similar defect types (P_b centers) and (interfacial) defect concentrations. In natural abundance silicon nanoparticles, the build-ups were found to be mono-exponential[1,2], which was explained by the fast spin diffusion with fast in this context referring to the time for bulk spin diffusion to spread across the nanoparticle radius taking only a small fraction of the experimental build-up time[1,2]. Hence, the hours-long hyperpolarization build-up needs to be governed by a different process, e.g. the spin diffusion across the hypershifted nuclei[1–4]. For the present ^{29}Si enriched particles, the two time constants of the bi-exponential build-up might reflect the spin diffusion across the hypershifted nuclei[1–4] and bulk spin diffusion for microparticles.

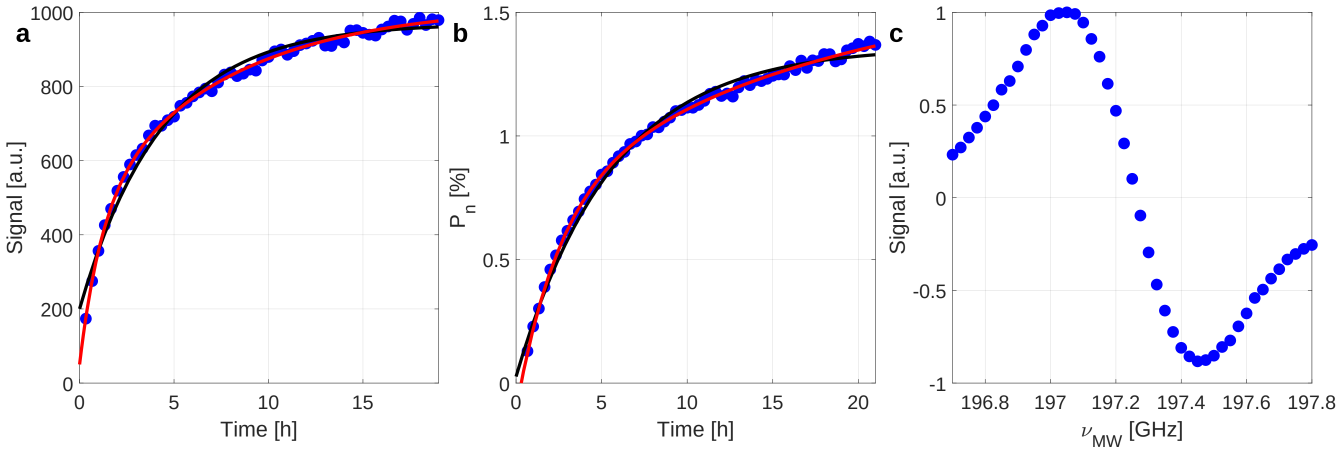

Figure S1. **(a)** Build-up of the ^{29}Si isotope enriched silicon particles at 3.4 T and 3.5 K. The black (red) lines refer to mono- (bi-) exponential fits to the data with build-up time constants of 4.4 ± 0.4 h (1.2 ± 0.4 h and 7.3 ± 1.8 h with $42\pm8\%$ of the signal from the fast component). **(b)** Build-up of the ^{29}Si isotope enriched silicon particles at 7 T and 3.5 K. The black (red) lines refer to mono- (bi-) exponential fits to the data with build-up time constants of 5.6 ± 0.4 h (2.5 ± 0.5 h and 18 ± 10 h with $48\pm4\%$ of the signal from the fast component). **(c)** DNP profile of the ^{29}Si isotope enriched silicon particles at 7 T and 3.5 K.

S3 Exponential fits of diamond

Fig. S2 compares mono-, bi- and stretched exponential fits for diamond build-up and decays at 3.4 T, 3.5 K. The R^2 and root mean square error (RMSE) of the three fits for build-up and decay are summarized in Tab. S1. The bi- and stretched exponential fits describe the experimental data visually and in terms of fit quality slightly better than the mono-exponential models, with all three performing reasonably.

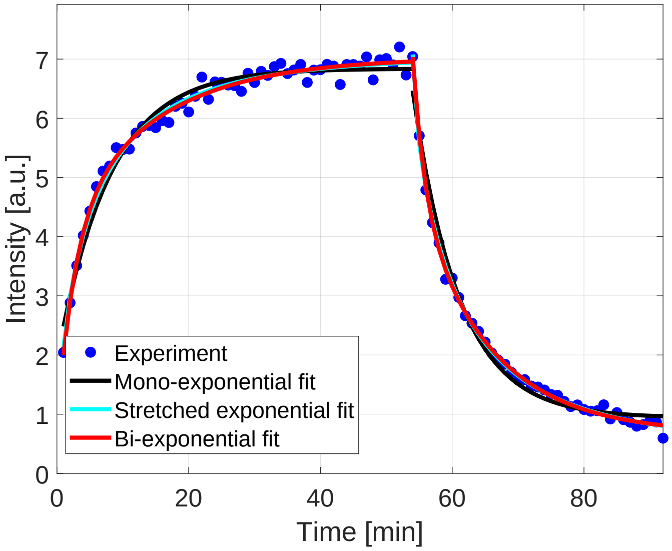

Figure S2. Measured build-up and decay at 3.4 T, 3.5 K of DNP in diamond. The build-up and decay are each fitted with a mono-exponential ($A\cdot e^{-t/\tau}$), bi-exponential ($A\cdot(\alpha e^{-t/tau_1}+(1-\alpha)e^{-t/\tau_2})$) and stretched exponential ($A\cdot e^{-(t/\tau)^{\beta}}$) model. The stated models are for the decay case and can be straightforwardly adjusted for the build-up case, e.g. ($A\cdot(1-e^{-t/\tau})$) for the mono-exponential build-up. The R^2 and RMSE of the fits are given in Tab. S1. The different fit models for diamond were first compared in the Supplementary Information of Ref.[5] for the same diamond sample as in this work but at 7 T, 3.4 K.

Since for the data in Fig. 2a of the main text only two measurements are available per shown data point (before and after shuttling), stretched and bi-exponential models have the same or a larger number of parameters than experimental data points. In a similar direction, the fits in Fig. 2c of the main text rely on three (10 K, 75 mT) and four (3.6 K, 200 mT) experimental data points. This would only allow to use a stretched exponential model in Fig. 2c of the main text. However, comparing T_1 times

	Build-up			Decay		
	Mono-exp.	Stretched exp.	Bi-exp.	Mono-exp.	Stretched exp.	Bi-exp.
R^2	0.973	0.985	0.987	0.985	0.997	0.998
RMSE	18.2	13.4	12.8	18.1	7.6	7.6

Table S1. R^2 and root mean square error (RMSE) of the mono-, stretched and bi-exponential build-up and decay fits from Fig. S2.

from mono- and stretched exponential fits would complicate any interpretation. In addition, from our experience, stretched exponential fits require a high-quality build-up or decay (enough points without much noise) to yield a stable combination of time constant and stretch parameter.

S4 DNP in diamond

Recent EPR experiments identified another diamond defect that appears essential for DNP involving P1 centers[5–7]. This additional broad EPR signal was attributed to clustering of P1 centers[6,7] or alternatively as another known defect in diamond, e.g. N2 or N3 centers[5].

While the idea of clusters of P1 centers being essential for the EPR and DNP properties of diamond is interesting, there are several weaknesses in this hypothesis. (i) At room temperature, the EPR spectra are symmetric around the $m_I = 0$ line at 6.9-13.8 T[5,7,8]. In contrast, the EPR spectra become asymmetric upon cooling[5,8]. The asymmetry is directly visible to the eye and has been measured in different samples by different groups at different fields[5,8]. It is difficult to imagine how such an asymmetry should arise from the proposed P1 cluster models and the asymmetry being a measurement artifact appears unlikely[5]. (ii) P1 centers might only be a minority fraction of the total nitrogen concentration, e.g. in Ref.[7] the sample contained ~ 20 ppm of P1 centers and a nitrogen concentration below 200 ppm. The fate of the remaining nitrogen content is not discussed although previous work (directly) observed that several nitrogen defects overlap with the EPR lines of P1 centers[9–13] (iii) Electronic $T_{1,\mathrm{e}}$ of samples containing only P1 centers (25 or 95 ppm) range from seconds to hundreds of seconds below 10 K[14]. Such long electronic relaxation times appear incompatible with nuclear hyperpolarization build-up times of ≤ 12 min, considering that each defect on average needs to hyperpolarize a few hundred nuclei[5].

N2 or N3 centers could shorten the electronic relaxation time of the P1 centers[14] or might be directly involved in the DNP and relaxation processes[5]. While the P1 centers have a negligible g-factor anisotropy, the N3 center is reported to have a small g-factor anisotropy and hyperfine couplings smaller than 5 MHz[11]. Hence, the N3 electron spectral line shape is mostly given by the g-factor anisotropy and, for low magnetic fields, would appear as multiple closely spaced lines with decreasing line width for decreasing field strength. Therefore, if N3 centers or another defect are involved in the DNP and relaxation of diamond, a g-factor anisotropy would be encountered as required for Eq. 2 of the main text. We emphasize that the proposed explanation of the linear field dependence requires further study in terms of understanding the spin system in diamond as well as quantitative agreement between relaxation calculations and experiment.

References

1. von Witte, G. *et al.* Controlled synthesis and characterization of porous silicon nanoparticles for dynamic nuclear polarization. *Nanoscale* **16**, 19385–19399, DOI: 10.1039/D4NR02603A (2024).

2. von Witte, G. *et al.* The role of nuclear spin diffusion in dynamic nuclear polarization of crystalline nanoscale silicon particles, DOI: 10.48550/arXiv.2412.10536 (2024). ArXiv:2412.10536 [quant-ph].

3. von Witte, G., Kozerke, S. & Ernst, M. Two-electron two-nucleus effective Hamiltonian and the spin diffusion barrier. *Sci. Adv.* **11**, eadr7168, DOI: 10.1126/sciadv.adr7168 (2025).

4. Redrouthu, V. S. *et al.* Overcoming the nuclear spin diffusion barrier in dynamic nuclear polarization via electron-electron flip-flop. *Phys. Rev. B* **111**, 104417, DOI: 10.1103/PhysRevB.111.104417 (2025). Publisher: American Physical Society.

5. von Witte, G. *et al.* Temperature-Dependent Dynamic Nuclear Polarization of Diamond. *The J. Phys. Chem. C* DOI: 10.1021/acs.jpcc.5c02747 (2025). Publisher: American Chemical Society.

6. Bussandri, S. *et al.* P1 Center Electron Spin Clusters Are Prevalent in Type Ib Diamonds. *J. Am. Chem. Soc.* **146**, 5088–5099, DOI: 10.1021/jacs.3c06705 (2024). Publisher: American Chemical Society.

7. Nir-Arad, O., Shlomi, D. H., Manukovsky, N., Laster, E. & Kaminker, I. Nitrogen Substitutions Aggregation and Clustering in Diamonds as Revealed by High-Field Electron Paramagnetic Resonance. *J. Am. Chem. Soc.* **146**, 5100–5107, DOI: 10.1021/jacs.3c06739 (2024). Publisher: American Chemical Society.

8. Stern, Q. *et al.* P1 center network in high-pressure high-temperature diamonds is a readily accessible source of nuclear hyperpolarization at 14 T, DOI: 10.26434/chemrxiv-2025-3r9qt (2025).

9. Loubser, J. H. & Van Wyk, J. A. Electron spin resonance in the study of diamond. *Reports on Prog. Phys.* **41**, 1201–1248, DOI: 10.1088/0034-4885/41/8/002 (1978).

10. van Wyk, J. A. Carbon-12 hyperfine interaction of the unique carbon of the P2 (ESR) or N3 (optical) centre in diamond. *J. Phys. C: Solid State Phys.* **15**, L981, DOI: 10.1088/0022-3719/15/27/007 (1982).

11. van Wyk, J. A., Loubser, J. H. N., Newton, M. E. & Baker, J. M. ENDOR and high-temperature EPR of the N3 centre in natural type Ib diamonds. *J. Physics: Condens. Matter* **4**, 2651, DOI: 10.1088/0953-8984/4/10/027 (1992).

12. Terblanche, C. J., Reynhardt, E. C., Rakitianski, S. A. & Van Wyk, J. A. 13 C Spin–Lattice Relaxation in Natural Diamond: Zeeman Relaxation in Fields of 500 to 5000 G at 300 K Due to Fixed Paramagnetic Nitrogen Defects. *Solid State Nucl. Magn. Reson.* **19**, 107–129, DOI: 10.1006/snmr.2001.0025 (2001).

13. Terblanche, C. J., Reynhardt, E. C. & van Wyk, J. A. 13C Spin–Lattice Relaxation in Natural Diamond: Zeeman Relaxation at 4.7 T and 300 K Due to Fixed Paramagnetic Nitrogen Defects. *Solid State Nucl. Magn. Reson.* **20**, 1–22, DOI: 10.1006/snmr.2001.0026 (2001).

14. Reynhardt, E. C., High, G. L. & van Wyk, J. A. Temperature dependence of spin-spin and spin-lattice relaxation times of paramagnetic nitrogen defects in diamond. *The J. Chem. Phys.* **109**, 8471–8477, DOI: 10.1063/1.477511 (1998).
